# Transition from acute kidney injury to chronic kidney disease in a long-term murine model of Shiga toxin-induced hemolytic-uremic syndrome

**DOI:** 10.3389/fimmu.2024.1469353

**Published:** 2024-10-10

**Authors:** Jamila Wegener, Sophie Dennhardt, Ivonne Loeffler, Sina M. Coldewey

**Affiliations:** ^1^ Department of Anesthesiology and Intensive Care Medicine, Jena University Hospital, Jena, Germany; ^2^ Septomics Research Center, Jena University Hospital, Jena, Germany; ^3^ Department of Internal Medicine III, Jena University Hospital, Jena, Germany; ^4^ Center for Sepsis Control and Care (CSCC), Jena University Hospital, Jena, Germany

**Keywords:** chronic kidney disease, acute kidney injury, animal model, fibrosis, anemia, hemolytic-uremic syndrome

## Abstract

**Introduction:**

Up to 40% of patients with typical hemolytic–uremic syndrome (HUS), characterized by microangiopathic hemolytic anemia and acute kidney injury (AKI), develop long-term consequences, most prominently chronic kidney disease (CKD). The transition from AKI to CKD, particularly in the context of HUS, is not yet fully understood. The objective of this study was to establish and characterize a Shiga toxin (Stx)-induced long-term HUS model to facilitate the study of mechanisms underlying the AKI-to-CKD transition.

**Methods:**

C57BL/6J mice were subjected to 5, 10, 15, or 20 ng/kg Stx on days 0, 3, and 6 of the experiment and were sacrificed on day 14 or day 21 to identify the critical time of turnover from the acute to the chronic state of HUS disease.

**Results:**

Acute disease, indicated by weight loss, plasma neutrophil gelatinase-associated lipocalin (NGAL) and urea, and renal neutrophils, diminished after 14 days and returned to sham level after 21 days. HUS-associated hemolytic anemia transitioned to non-hemolytic microcytic anemia along with unchanged erythropoietin levels after 21 days. Renal cytokine levels indicated a shift towards pro-fibrotic signaling, and interstitial fibrosis developed concentration-dependently after 21 days. While Stx induced the intrarenal invasion of pro-inflammatory M1 and pro-fibrotic M2 macrophages after 14 days, pro-fibrotic M2 macrophages were the dominant phenotype after 21 days.

**Conclusion:**

In conclusion, we established and characterized the first Stx-induced long-term model of HUS. This tool facilitates the study of underlying mechanisms in the early AKI-to-CKD transition following HUS and allows the testing of compounds that may protect patients with AKI from developing subsequent CKD.

## 1 Introduction

More than every 10th human worldwide develops chronic kidney disease (CKD) during life and the number is increasing rapidly (1). CKD is defined as abnormalities in the kidney structure or function that persist for more than three months, with implications for health. It is identified by damage markers, e.g., albumin excretion and/or a glomerular filtration rate (GFR) of <60 mL/min/1.73 m^2^ (2). In contrast to most non-communicable and communicable diseases, CKD mortality increased drastically in the past years, and according to the Global Burden of Disease (GBD) study, it belongs to the top 10 leading causes of death in the elderly population (3). Besides age, risk factors influencing the prevalence of CKD are sex, ethnicity, and comorbidities (4).

The highest number of patients with CKD caused by a preceding disease are suffering from diabetes mellitus. However, other diseases can be prone to long-term consequences and CKD development such as the hemolytic–uremic syndrome (HUS) (4, 5). HUS is a systemic complication mainly induced by the infection with Shiga toxin (Stx)-producing *Escherichia coli* (STEC). The acute disease preferentially affecting children of young age (<5 years) is induced by the ribotoxic function of the STEC main virulence factor Stx (6). Through the high level of its receptor in glomerular microvascular endothelial cells, Stx induces inhibition of protein biosynthesis and thus damage of the vascular endothelium and cell death predominantly in the kidney (7). This results in the symptomatic triad of thrombocytopenia, hemolytic anemia, and acute kidney injury (AKI).

Although the mortality of HUS is low, up to 40% of patients develop long-term consequences with the majority developing CKD (5). Even patients with mild HUS and no need for dialysis can develop CKD more than 10 years after initial acute disease. Alconcher and colleagues recently published a long-term follow-up study of undialyzed STEC-HUS patients, where they stated that asymptomatic patients without proteinuria or hypertension can develop CKD 1 year after acute stage, pointing out that follow-up control and support is crucial (8).

Several pathologic mechanisms have been linked to the progression of AKI to CKD although the transition is complex and not fully understood (9). At the tissue level, the development of fibrosis is a key factor in the progression to CKD. Upon maladaptive repair, myofibroblasts can originate from different sources—from proliferating resident fibroblasts, pericytes detaching from their perivascular site, and from endothelial (EndoMT) and from epithelial (EMT)-to-mesenchymal transition. Myofibroblasts persist in the kidneys, progressively deposit extracellular matrix (ECM) molecules and consequently induce fibrosis, capillary rarefaction, and chronic inflammation (10, 11). The involvement of resident macrophages and recruitment of other leukocytes and their immunological cross-talk with endo- and epithelial cells is crucial in both AKI and CKD (11). Diverse functions of macrophages are highly dependent on their phenotype distinguishing the M1 pro-inflammatory and M2 anti-inflammatory and pro-fibrotic phenotype (12). M2 macrophages highly express cytokines driving fibrosis, e.g., transforming growth factor-beta (TGF-β), in an even higher extent than myofibroblasts, discovered in a CKD model induced by unilateral ureteral obstruction (UUO), suggesting macrophages as the major source (13, 14).

Several preclinical models have been established in the research of renal injury and repair to improve the understanding of CKD pathogenesis. Most common rodent models to mimic CKD development are based on UUO, ischemia–reperfusion injury (IRI), or 5/6 nephrectomy. These models rely on the renal obstruction of blood and thereby oxygen and nutrient supply inducing damage and fibrosis to mimic operational complications. CKD in rodents can also be initiated by nephrotoxicity through, e.g., cisplatin or folic acid, or by the stimulation of the host immune system by lipopolysaccharide (LPS), but with high risk of early death (15, 16).

Hitherto, no long-term model of CKD progression through HUS exists and long-term clinical studies are extremely rare due to high rates in loss of follow-ups. Consequently, mechanisms of AKI-to-CKD transitions are still poorly understood.

We aim to establish a CKD model induced by bacterial toxemia to study the progression of long-term effects in experimental HUS. We mimic the physiological transition from an acute HUS disease to a chronic state in mice associated with recovered overall appearance, persisting non-hemolytic anemia, pro-fibrotic signaling and immune phenotype as well as interstitial fibrosis.

## 2 Material and methods

### 2.1 Experimental HUS

Fifty male 10- to 12-week-old C57BL/6J wild-type mice were randomly assigned, by the random pick of one out of two prepared treatment plans, to the following groups: sham, 5 ng/kg Stx, 10 ng/kg Stx, 15 ng/kg Stx, and 20 ng/kg Stx. Stx from the same stock used in previous experiments (17) purified from an O157:H7 enterohemorrhagic *E. coli* (EHEC) strain 86-24 patient isolate was used to induce experimental HUS. Five, 10, 15, or 20 ng/kg body weight Stx or 0.9% saline (sham) was injected intravenously on days 0, 3, and 6 of the experiment and mice were monitored three times per day according to the previously described HUS scoring system (17) along with 800 µL of subcutaneous administration of Ringer’s lactate solution for volume replacement. Mice of each group were sacrificed on either day 14 or day 21 after initial Stx injection (day 0). Thereby, the number of individuals implies five mice in each group according to Mayer and Muche’s recommendations (18). Mice were bred in-house under specific pathogen-free conditions in individual ventilated cages (IVCs). For acclimatization phase 1 week before the experiments and during the experimental procedures, mice were kept in open cages with bedding material and tubes as environmental enrichment and under standardized laboratory conditions (22 ± 1°C, 55% ± 10% humidity) with free access to standard rodent chow and water. All treatments and scorings were carried out blinded in consistent order and in a separate room to minimize stress. All animal experiments were approved by the regional animal welfare committee and the Thuringian State Office for Consumer Protection (registration number UKJ-20-018) and were performed in accordance with German legislation and the approved guidelines as well as in compliance with the ARRIVE guidelines. Outcome assessment comparing all groups were carried out blinded and allocations were corrected before statistical analysis.

### 2.2 Whole blood and plasma analysis

Whole blood sampling and plasma preparation were carried out as described previously (17). Hemograms were measured immediately after collection using the hematology device scil Vet abc Plus+ (scil animal care company GmbH). Neutrophil gelatinase-associated lipocalin (NGAL), urea, erythropoietin (EPO), ferritin, hepcidin, and lactate dehydrogenase (LDH) activity were determined in plasma of mice using commercial kits according to the manufacturer’s protocol (**Supplementary Table S1**).

### 2.3 Histology and immunohistochemistry

Fresh kidney halves were fixed in 5% buffered formaldehyde solution (Fischar, Germany), dehydrated in descending alcohol solutions and embedded in paraffin (Thermo Fisher Scientific, USA) as previously described (17). FFPE sections of 2 µm thickness were prepared to receive renal cross-sections including areas of cortex and medulla. AZAN after Heidenhain (Morphisto, Germany) staining was performed according to the manufacturer’s instructions. For immunohistochemistry, sections were hydrated using descending alcohol solutions, blocked in 3% hydrogen peroxide (Carl Roth GmbH + Co. KG, Germany), and epitope retrieval was carried out at 110°C for 10 min in target retrieval solution (Dako, Denmark). 1× Tris buffer (10× Tris: 0.5 M Tris, 3 M NaCl, and HCl 37% dropwise until pH 7.6; 1% Tween20) was used as washing buffer. Unspecific antibody binding sites were blocked using bovine serum albumin (BSA; Sigma Aldrich, USA), serum (GE Healthcare, USA), and avidin/biotin solutions (Vector Laboratories, USA). Renal sections were incubated in primary antibody (**Supplementary Table S2**) at 4°C overnight. The next day, sections were incubated in secondary antibody (**Supplementary Table S3**); specificity was enhanced by VECTASTAIN Elite ABC HRP Kit (Vector Laboratories, USA) for 30 min each and ImmPACT DAB Peroxidase (HRP) Substrate (Vector Laboratories, USA) was added. Nuclei were counterstained with hematoxylin (Carl Roth GmbH + Co. KG, Germany). Quantification of Ly6G level was performed as described previously (19). Intersitital fibrosis was quantified counting AZAN positive caskets (blue staining) in a grid area (10 × 10 caskets; grid area: 0.097 mm²) for 20 adjacent cortical areas (magnification 400×). CD206 and CD86 abundance was quantified by counting positive grid intersections for 20 adjacent cortical areas (magnification 400×) using the grid mentioned above. Images were taken with the KEYENCE BZ-X800 microscope and BZ-X800 viewer after white balance and auto exposure at a magnification of 400×.

### 2.4 Immunoblotting

Cross-sections of frozen kidneys (20–30 mg), including tissue of renal cortex and medulla, were homogenized in lysis buffer [1% IGEPAL^®^ CA-630 (Sigma Aldrich, USA), 50 mM Tris-aminomethan (TRIS), pH 8.0 (Carl Roth GmbH + Co. KG, Germany), 150 mM NaCl (Carl Roth GmbH + Co. KG, Germany), 5 mM NaF (Sigma Aldrich, USA), 1 mM Na_3_VO_4_ (Sigma Aldrich, USA), and protease inhibitor cocktail 1:100 (Roche Holding, Switzerland)]. After centrifugation for 10 min at 14,000 × *g*, the total protein amount of the supernatant was determined using Pierce Detergent Compatible Bradford Assay Kit (Thermo Fisher Scientific, USA). Total protein (40 µg) was reduced in Laemmli buffer at 95°C for 5 min and loaded onto 10% TGX Stain-Free FastCast gels (Bio-Rad Laboratories, USA). Transfer, blocking, antibody incubation, and signal development were carried out as described previously (19) using anti-TGF-β (Cell Signaling Technologies, UK, #3711S) and anti-rabbit IgG, HRP-linked antibody (Cell Signaling Technologies, UK, #7074S). To quantify, the Image Lab software was used to normalize abundance of protein of interest to total lane protein visualized by stain-free technology (Bio-Rad Laboratories, USA) instead of a reference protein for loading control (20). When normalization factors exceeded 1.3 or fell below 0.7, bands were excluded from calculations.

### 2.5 Erythropoietin assessment and cytokine multiplex analysis

Renal tissue lysates were prepared fresh as described in section immunoblotting. Renal erythropoietin (EPO) was determined using a commercial kit according to the manufacturer’s protocol (**Supplementary Table S1**). Total protein (500 µg) was used to measure cytokines using the Bio-Plex Pro Mouse Chemokine 31-Plex panel assay (Bio-Rad, USA) according to the manufacturer’s instructions.

### 2.6 Statistics

Data were statistically analyzed using GraphPad Prism 7.05 (GraphPad Software, USA) and are presented as mean standard deviation (SD) for *n* observations (animals). Gaussian distribution was tested using the Shapiro-Wilk normality test at a significance level of 0.05. When data were normally distributed, unpaired *t*-test was used to determine differences comparing two groups, one-way ANOVA followed by Sidak’s *post-hoc* test was used to determine differences comparing more than two groups, or two-way ANOVA followed by Dunnett’s *post-hoc* test was used to determine differences when all groups were compared, with time and concentration as factors. When data were not normally distributed, Mann–Whitney *U* test was used to determine differences comparing two groups. Furthermore, values that were three times the SD of the corresponding group were tested for outliers using the ROUT method with Q = 1% and were excluded.

## 3 Results

### 3.1 Acute response to experimental HUS attenuates after 21 days

**Figure 1:**
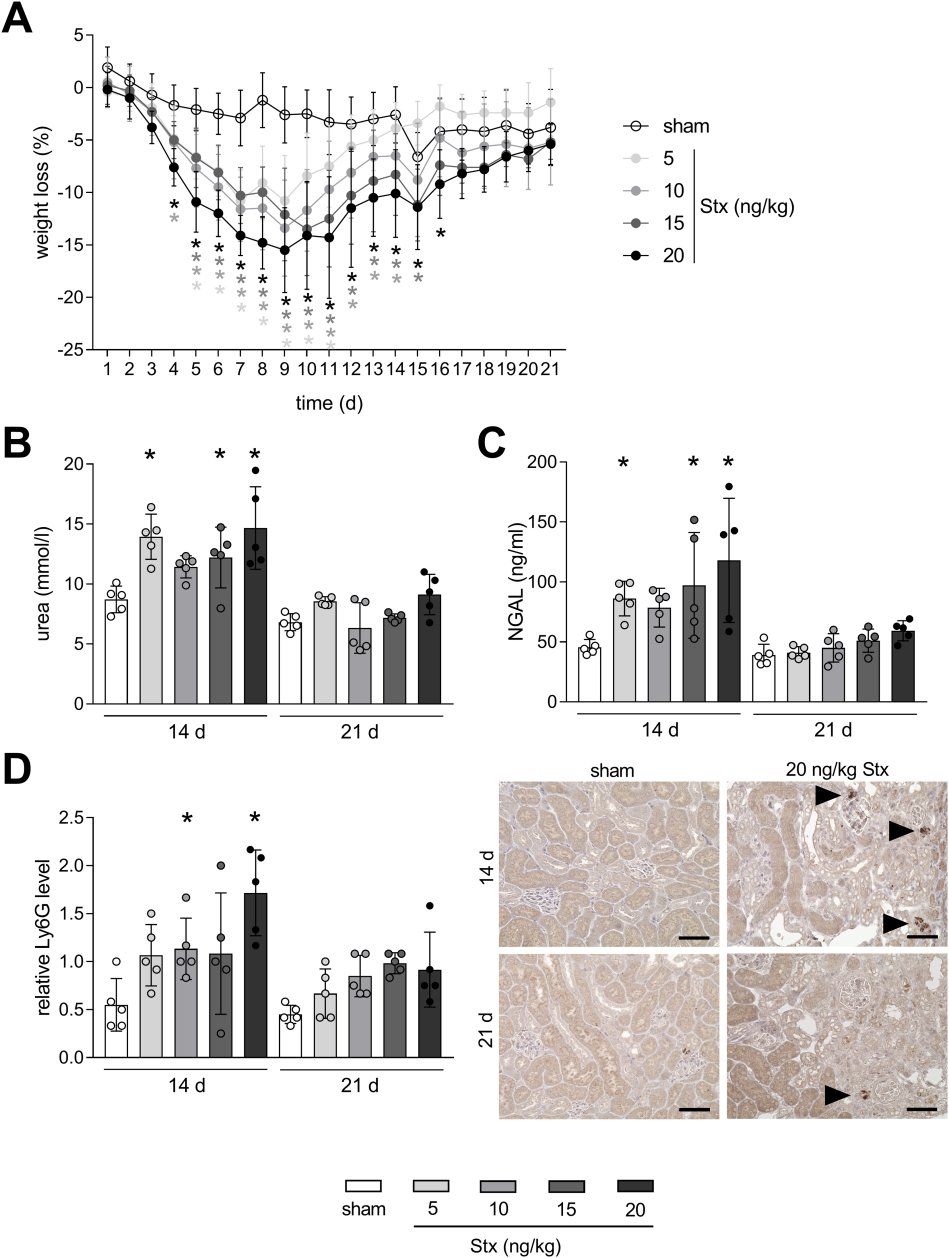
Time-dependent mitigation of acute phase response in mice with experimental hemolytic–uremic syndrome (HUS). **(A)** Weight loss of sham mice or mice with experimental HUS [depicted Shiga toxin (Stx) concentrations] over the course of the experiment. Plasma levels of **(B)** urea and **(C)** neutrophil gelatinase-associated lipocalin (NGAL) in sham mice or mice with experimental HUS (depicted Stx concentrations) 14 or 21 days after HUS induction. **(D)** Quantification and representative images (for 20 ng/kg Stx only) of relative lymphocyte antigen 6 family member G (Ly6G) abundance, representing neutrophils, in renal tissue of sham mice or mice with experimental HUS (depicted Stx concentrations) 14 or 21 days after HUS induction. Bar = 50 µm. Arrows indicate Ly6G^+^ neutrophils. Data are shown as mean ± SD. **(A)** *n* = 5–10. Values were combined for the first 14 days from the 14-day and 21-day group. **(B–D)** *n* = 5. *p < 0.05 compared to the corresponding sham group (two-way ANOVA and Dunnett’s multiple comparison test).

To analyze disease progression phenotypically, weight loss was assessed in mice for a time period of 21 days after HUS induction. Mice with experimental HUS developed a concentration-dependent weight loss compared with sham mice starting from day 4 (**Figure 1A**), which is already known from our well-established 7-day HUS study (17). Average weight loss peaked on day 9 after HUS induction, from which mice gained weight again. Weight loss on day 14 was still significantly higher in mice treated with 10, 15, and 20 ng/kg Stx compared to sham mice. However, the weight of mice with experimental HUS returned to sham level 21 days after HUS induction. Urea and NGAL, both known to be elevated in plasma in the early phase of renal injury, were increased in plasma 14 days after HUS induction, but reduced compared to 7-day HUS mice (19), and returned to sham level 21 days after HUS induction (**Figures 1B, C**). Neutrophils, which represent first-line defender cells and play a pivotal role in the pathology of HUS, had migrated into renal tissue 14 days after HUS induction in mice that received 10 and 20 ng/kg Stx compared to sham mice as indicated by the murine surface marker lymphocyte antigen 6 family member G (Ly6G, **Figure 1D**). Twenty-one days after HUS induction, renal neutrophil abundance was comparable to sham mice, independent on the applied Stx concentration. Taken together, weight assessment, renal tissue damage parameter, and acute-phase immune cells in renal tissue indicate that mice recover from the acute HUS disease 21 days after HUS induction.

**Figure 2:**
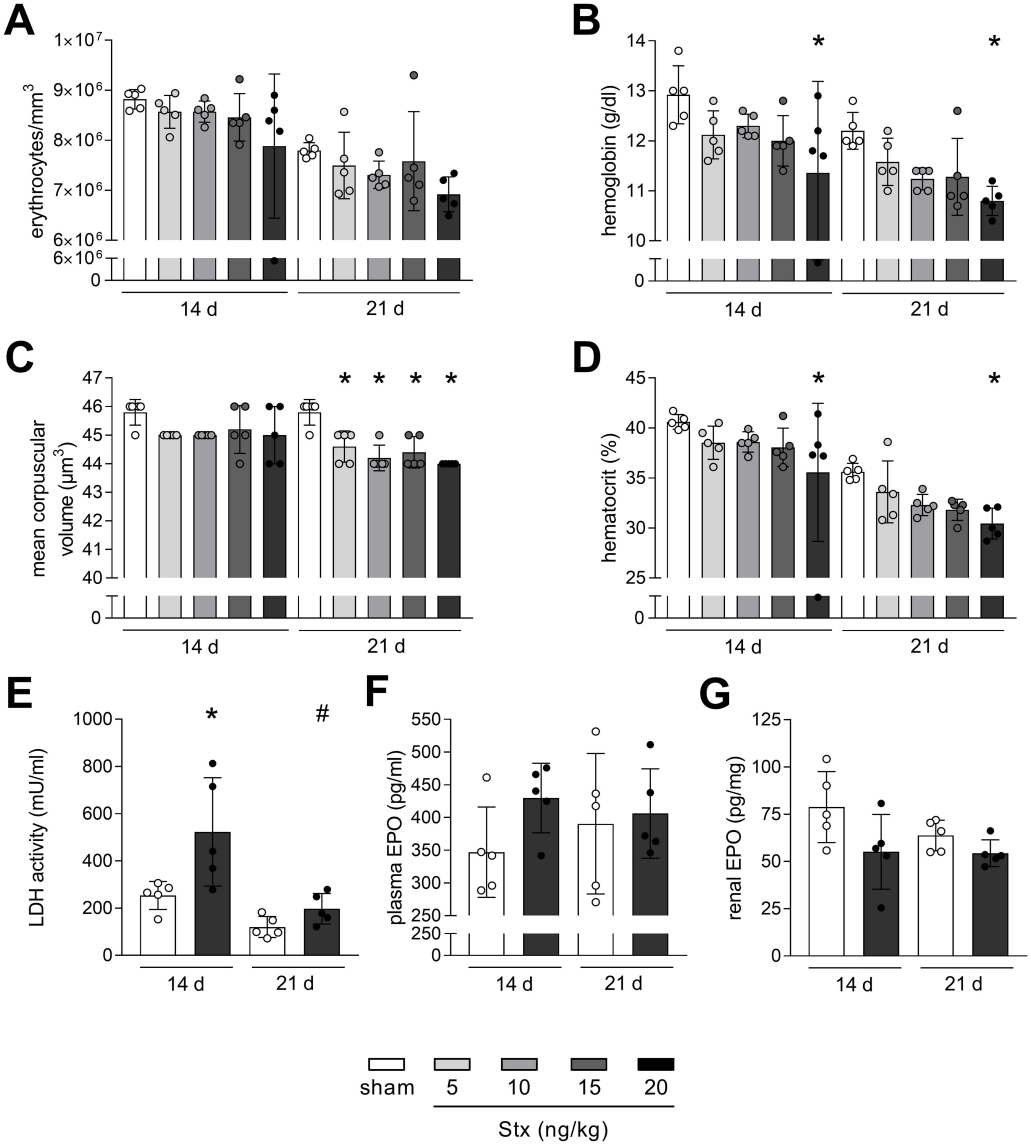
Non-hemolytic anemia in mice with experimental hemolytic–uremic syndrome (HUS). **(A)** Erythrocytes, **(B)** hemoglobin, **(C)** mean corpuscular volume (MCV), and **(D)** hematocrit in whole blood of sham mice or mice with experimental HUS [depicted Shiga toxin (Stx) concentrations] 14 or 21 days after HUS induction. **(E)** LDH activity in plasma of sham mice or mice with experimental HUS (20 ng/kg Stx) 14 or 21 days after HUS induction. EPO levels measured in **(F)** plasma and **(G)** renal tissue of sham mice or mice with experimental HUS (20 ng/kg Stx) 14 or 21 days after HUS induction. Data are shown as mean ± SD. *n* = 5. **(A–D)** *p < 0.05 compared to the corresponding sham group (two-way ANOVA and Dunnett’s multiple comparison test). **(E–G)** *p < 0.05 compared to the corresponding sham group and #*p* < 0.05 14 days 20 ng/kg Stx vs. 21 days 20 ng/kg Stx (one-way ANOVA and Sidak’s multiple comparison test).

### 3.2 Anemia persists in mice with experimental HUS after 21 days but without hemolysis

Hemolytic anemia is a central symptom of HUS. Therefore, parameters reflecting anemia and hemolysis were analyzed in mice 14 and 21 days after HUS induction. Erythrocytes were reduced in mice with experimental HUS 14 and 21 days after HUS induction, but without statistical significance (**Figure 2A**). Hemoglobin and hematocrit levels were both significantly decreased in mice with experimental HUS, when exposed to 20 ng/kg Stx, 14 and 21 days after HUS induction (**Figures 2B, D**). The mean corpuscular volume of erythrocytes was significantly decreased in mice 21 days after HUS induction, irrespective of the applied Stx concentration (**Figure 2C**). EPO level and LDH activity were only measured in mice exposed to the highest Stx concentration based on significantly altered hematocrit and hemoglobin levels. LDH activity in plasma, indicating hemolysis, was increased in HUS mice only 14 days but not 21 days after HUS induction (**Figure 2E**), whereas EPO levels in plasma and renal tissue were unchanged, 14 and 21 days after HUS induction (**Figures 2F, G**). Plasma levels of hepcidin and ferritin, which are involved in resorption and storage of iron, were slightly increased 14 days after HUS induction (hepcidin: with 15 ng/kg and 20 ng/kg, ferritin: with 20 ng/kg) and returned to sham level 21 days after HUS induction (**Supplementary Figure S1**). Overall, HUS mice exposed to 20 ng/kg Stx develop anemia that transitions from hemolytic to non-hemolytic 21 days after HUS induction, without impaired EPO production, unchanged hepcidin or ferritin levels, but lowered mean corpuscular volume of erythrocytes.

### 3.3 Renal fibrosis develops in mice with HUS after 21 days

**Figure 3:**
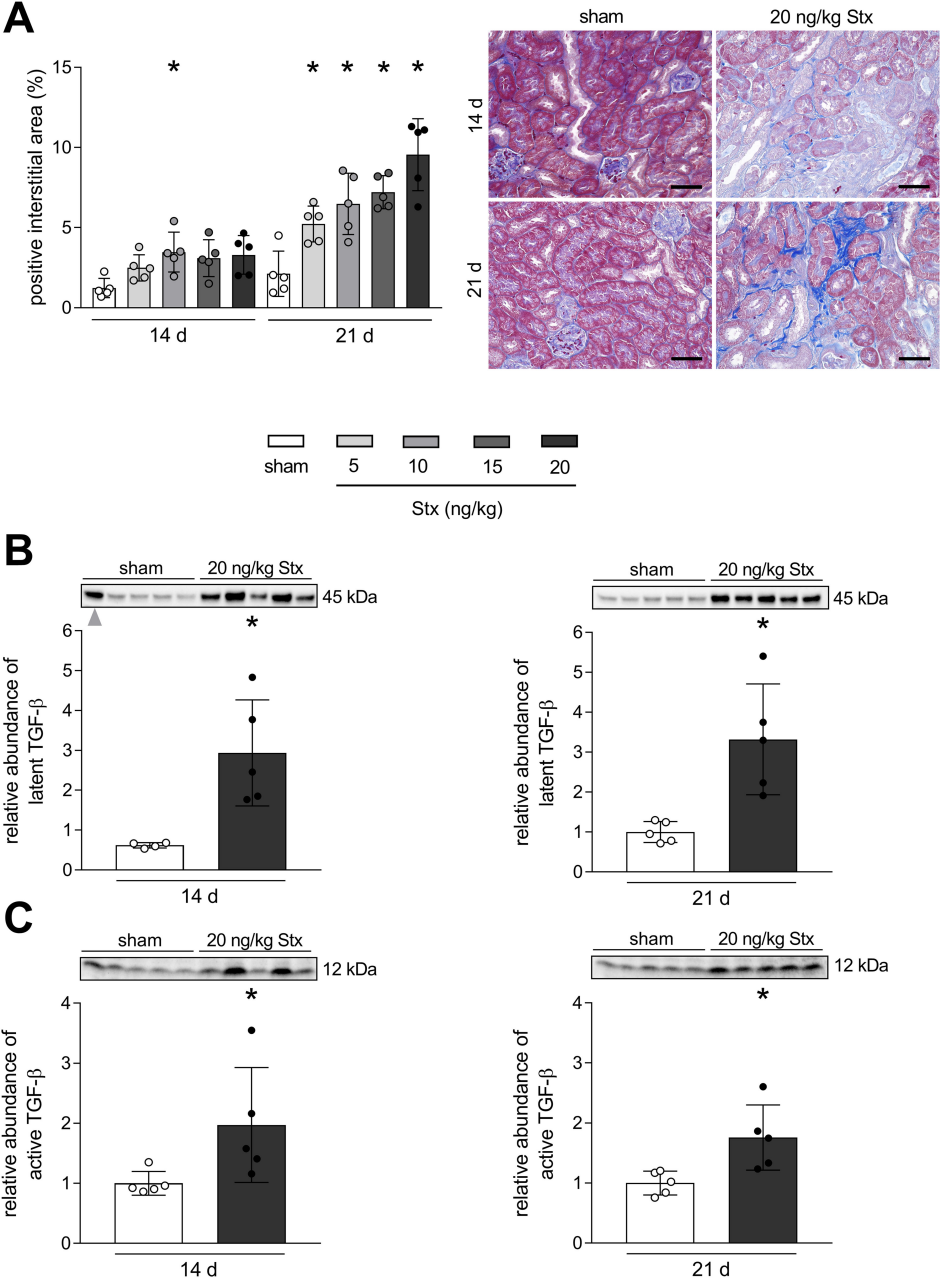
Development of renal fibrosis in mice with experimental hemolytic–uremic syndrome (HUS). **(A)** Quantification and representative images of AZAN after Heidenhain staining in renal tissue of sham mice or mice with experimental HUS [depicted Shiga toxin (Stx) concentrations] 14 or 21 days after HUS induction. Bar = 50 µm. Relative expression of **(B)** latent transforming growth factor-β (TGF-β) and **(C)** TGF-β active monomer in renal tissue of sham mice or mice with experimental HUS (20 ng/kg Stx) 14 or 21 days after HUS induction. Data are shown as mean ± SD. **(A)** *n* = 5. *p < 0.05 compared to the corresponding sham group (two-way ANOVA and Dunnett’s multiple comparison test). (**B**, left) *n* = 4. One outlier (gray arrow) was identified using the ROUT method with *Q* = 1% and was excluded from graph and statistics. (**B** right, **C**) *n* = 5. **(B, C)** *p < 0.05 compared to the corresponding sham group (Mann–Whitney *U* test).

In order to analyze whether Stx can induce long-term consequences in mice other than non-hemolytic anemia, the exacerbated deposition of ECM as a measure of fibrosis development was visualized in renal tissue by AZAN staining. The quantification of the positive interstitial area revealed that Stx induced a concentration-dependent increase in fibrosis 21 days after HUS induction (**Figure 3A**). At day 14 after HUS induction, renal fibrosis was only significantly apparent in mice exposed to 10 ng/kg Stx. The abundance of TGF-β, one of the key driver cytokines in the induction and progression of fibrosis, was analyzed in renal tissue lysates. Since fibrosis was most advanced in mice exposed to 20 ng/kg Stx 21 days after HUS induction, TGF-β levels were only measured in these mice and sham mice at both time points. Because of the unpredictable ribotoxic effects of Stx, stain-free technology instead of loading controls was used for the relative quantification of TGF-β (see Material and Methods). Stx induced an increase of the latent TGF-β as well as the active monomer 14 and 21 days after HUS induction (**Figures 3B, C**). This indicates an ongoing pro-fibrotic signaling cascade even 21 days after HUS induction.

### 3.4 Intrarenal macrophage switch 21 days after HUS induction from M1 to M2

**Figure 4:**
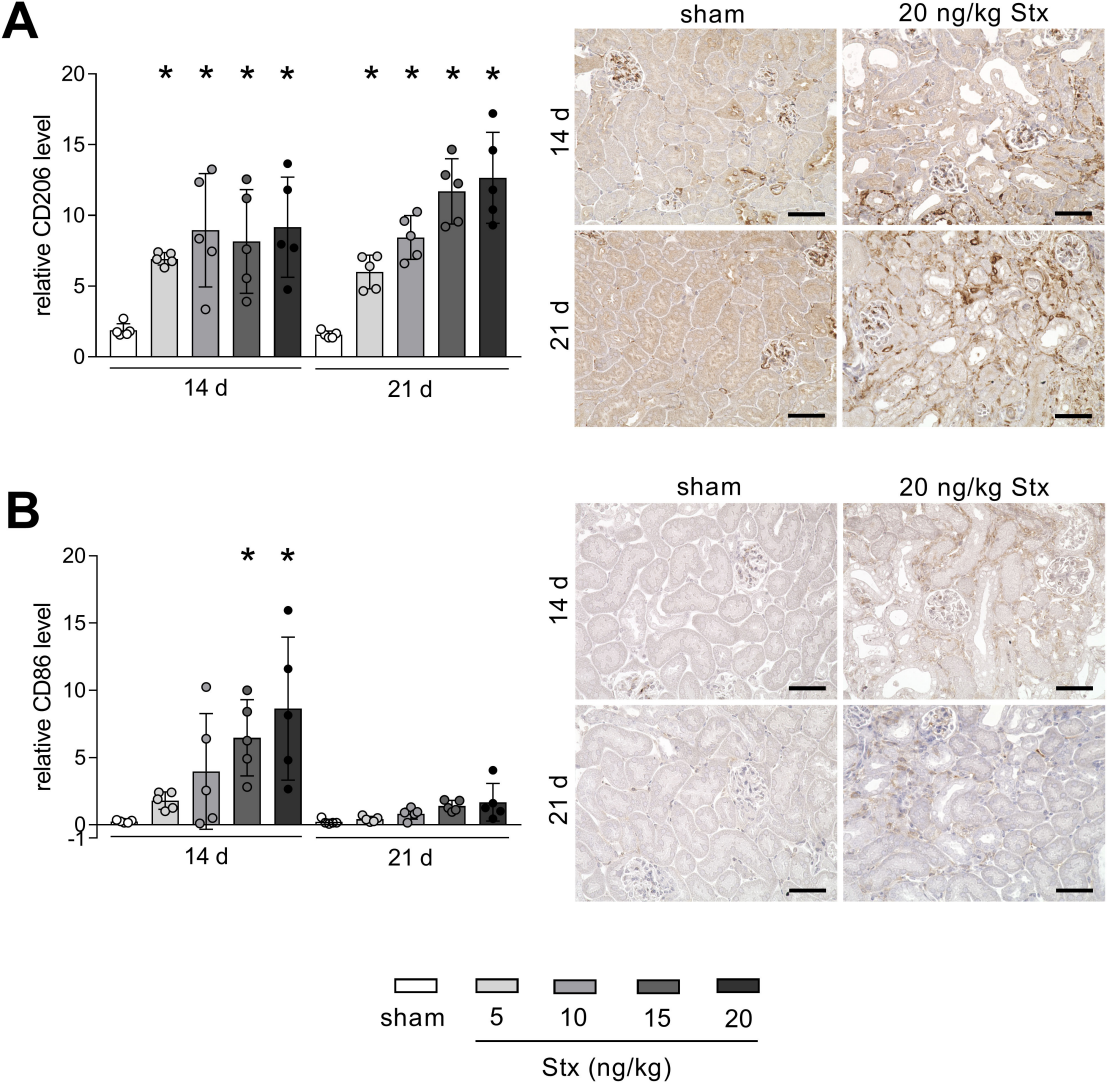
Renal abundance of macrophage marker of the M1 and M2 phenotype in mice with experimental hemolytic–uremic syndrome (HUS). Quantification and representative images [20 ng/kg Shiga toxin (Stx) only] of relative **(A)** CD206 and **(B)** CD86 abundance in renal tissue of sham mice or mice with experimental HUS 14 and 21 days after HUS induction (depicted Stx concentrations). Bar = 50 µm. Data are shown as mean ± SD. *n* = 5. *p < 0.05 compared to the corresponding sham group (two-way ANOVA and Dunnett’s multiple comparison test).

Dependent on the phenotype, macrophages secrete factors mitigating or driving renal fibrosis development. To characterize macrophages in renal tissue, markers for M1 pro-inflammatory phenotype (CD86) and M2 pro-fibrotic phenotype (CD206) were analyzed. At day 14 after HUS induction, macrophages of the M1 and M2 phenotype were present in renal tissue of mice with experimental HUS (**Figures 4A, B**). At day 21 after HUS induction, M1 macrophages were almost absent, whereas M2 macrophages remained highly abundant in a concentration-dependent manner (**Figures 4A, B**). To conclude, 14 days after HUS induction, M1 and M2 macrophages are present in the renal tissue of mice with experimental HUS, whereas 21 days after HUS induction, the predominant phenotypes are M2 macrophages, pointing towards a pro-fibrotic environment.

### 3.5 Intrarenal cytokine level changes 21 days after HUS induction

**Figure 5:**
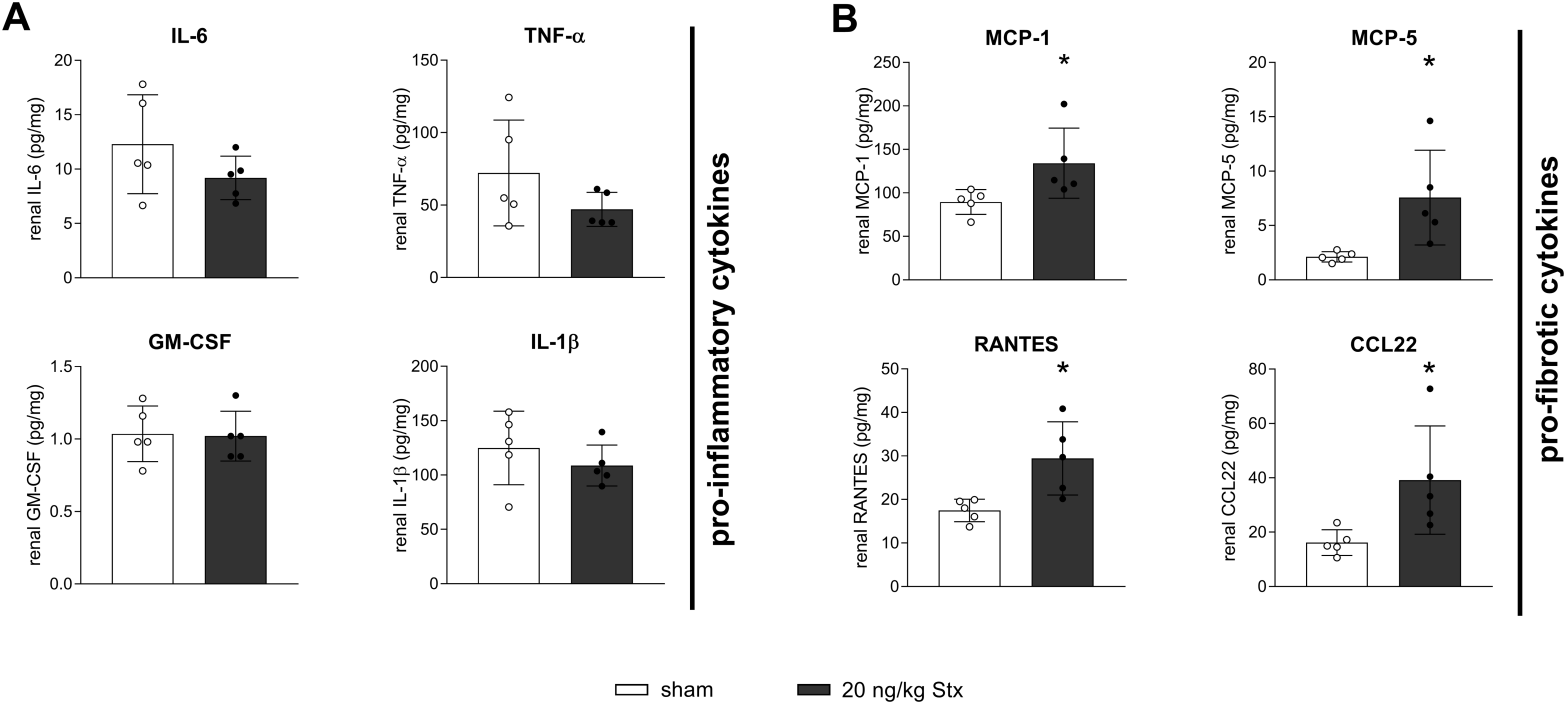
Renal cytokine signaling in mice with experimental hemolytic–uremic syndrome (HUS). Renal levels of **(A)** interleukin-6 (IL-6), tumor necrosis factor-α (TNF-α), interleukin-1β (IL-1β), granulocyte-macrophage colony-stimulating factor (GM-CSF), and **(B)** monocyte chemoattractant protein-1 (MCP-1), murine monocyte chemoattractant protein-5 (MCP-5), CC-chemokin-ligand-5 (RANTES), and CC-chemokine ligand 22 (CCL22) in sham mice or mice with experimental HUS 21 days after HUS induction [20 ng/kg Shiga toxin (Stx)]. Data are shown as mean ± SD. *n* = 5. *p < 0.05 compared to the corresponding sham group (IL-6, IL-1β, GM-CSF, MCP-1, MCP-5, RANTES, and CCL22: unpaired *t*-test, TNF-α: Mann–Whitney *U* test).

The acute phase of HUS is associated with and accelerated by excessive cytokine abundance in the kidney (21). A multiplex ELISA was performed on renal tissue lysates to detect the levels of 31 cytokines/chemokines. Because fibrosis and macrophage recruitment were concentration-dependent, only mice exposed to the highest Stx concentration were compared to sham mice. Besides the here mentioned cytokines/chemokines, all other cytokines/chemokines were unchanged compared to the sham group. HUS-associated pro-inflammatory cytokines and chemokines IL-6, IL-1β, GM-CSF, and TNF-α were unchanged in the renal tissue of mice 21 days after HUS induction compared to the sham group (**Figure 5A**). In contrast, concentrations of pro-fibrotic chemokines MCP-1, MCP-5, RANTES, and CCL22 were significantly higher in the renal tissue of mice 21 days after HUS induction compared to the sham group (**Figure 5B**). These chemokines especially attract macrophages, supporting the finding of increased macrophage infiltration into the kidneys of mice 14 and 21 days after HUS induction.

## 4 Discussion

With this work, we proved an onset of early CKD characteristics in mice 21 days after initial HUS induction. Renal interstitial fibrosis developed, along with associated signaling and subsequent macrophage phenotype, a diminished acute-phase and non-hemolytic anemia.

### 4.1 Acute disease declines after 21 days

Tissue regeneration after Stx-induced injury possibly explains recovered weight, systemic urea, and NGAL 21 days after HUS induction. Recent studies revealed that NGAL, elevated as early as 2 h after injury (22), is a strong predictor of CKD progression; however, plasma levels in patients with CKD compared to those with AKI are controversial (23). Neutrophils are the first leukocytes accumulating in the renal tissue upon AKI, also through HUS, and their number and activation status correlate with severity (24–26). Although neutrophils promote AKI progression and outcome, their involvement in CKD is not fully understood. The presence of neutrophils has been identified as an indicator of inflammation in CKD; however, the neutrophil-to-leukocyte ratio (NLR) alone could not predict CKD progression (27). In the present study, acute inflammation is decreased 21 days after HUS induction, indicating that neutrophils might be involved in the pathogenesis but not progression of CKD.

### 4.2 Recovered hemolysis but persistent anemia after 21 days

The symptomatic trials of HUS includes hemolytic anemia, also detected in our established model of subacute HUS (17). Here, we proved that hemolysis terminated 21 days after HUS induction, since plasma LDH levels declined to sham niveau. However, decreased levels of anemia markers hemoglobin and hematocrit 14 and 21 days after HUS induction with 20 ng/kg Stx indicate a persistent anemia even beyond 21 days. Anemia is a characteristic of CKD that correlates with severity and worsens prognosis (28). Multiple factors can lead to CKD anemia, such as reduced EPO production, EPO resistance, a low erythrocyte life span, and absolute or functional iron deficiency (28). We recently discovered elevated EPO levels in plasma of mice 7 days after HUS induction (29), whereas, here, EPO levels were unchanged in plasma and renal tissue 14 and 21 days after HUS induction. Interestingly, MCV was reduced 21 days after HUS induction, which is accompanied by low erythrocyte counts and/or low hemoglobin diagnostic for microcytic anemia, and the main cause is iron deficiency (30). In this case, although EPO production is normal, responsiveness is reduced due to the iron dependency of erythroblast-to-reticulocyte transition (31). Indicators of iron deficiency anemia besides erythrocyte counts, MCV, and hemoglobin are proteins of the iron metabolism (31). These include hepcidin and ferritin; however, we did not detect lowered plasma abundance of neither hepcidin nor ferritin 21 days after HUS induction, rather comparable to sham level. The pitfall of these proteins is their upregulation induced by inflammatory mediators, which we previously detected in plasma of mice with subacute HUS, for ferritin (32), and here for hepcidin and ferritin after 14 days.

### 4.3 Stx-induced renal damage leads to fibrosis, pro-fibrotic signaling, and macrophage phenotype switch

Repetitive damage to renal tissue can lead to CKD with inefficient blood perfusion, kidney function loss, and scarring, called fibrosis (33). Transitioned mesenchymal cells, pericytes, or activated tissue-resident fibroblasts excrete ECM molecules to an excessive and destructive extent (11). We detected interstitial fibrosis histologically by trichrome staining 21 days after HUS induction as the major proof of CKD development. The regulation of fibrogenesis is complex; however, TGF-β is known as the most important cytokine involved in the development and progression of CKD (33). In this study, we measured elevated levels of the latent as well as the active monomer of TGF-β in the kidneys of mice 14 and 21 days after HUS induction.

Macrophages are highly heterogeneous and subpopulation classification is based on their distinct function, origin, and tissue-specific location (34). Macrophages are referred to either classically activated M1 or alternatively activated M2 phenotype (34), identifiable by the distinct expression of cell surface markers such as CD86 primary on M1 and CD206 primary on M2 macrophages (35–38). The M1 phenotype is considered pro-inflammatory because of its capacity to express cytokines like IL-1, IL-6, and TNF-α, which contribute to leukocyte recruitment and activation. M2 macrophages express anti-inflammatory functions and are involved in tissue repair and fibrosis (34, 39). The profound presence of M2 macrophages in the renal tissue has been linked to defective healing and subsequent fibrosis in clinical and preclinical studies: In patients of diabetic nephropathy (DN), M2 macrophage abundance positively correlated with interstitial fibrosis, tubular atrophy and DN class (40). In biopsies of patients with new-onset immunoglobulin A (IgA) nephropathy, M2 macrophages were detected in sites of active fibrosis and cell numbers correlated with mesangial matrix expansion (41). Two further studies investigating renal allograft fibrosis discovered that high numbers of M2 macrophages correlated with alloimmune inflammation, injury, and fibrosis (42, 43). Along with clinical findings, several animal models highlight the role of M2 macrophages in fibrogenesis and progression. For instance, the depletion of M2 macrophages in a long-term model following IRI improved renal fibrosis, whereas adoptive transfer of M2c macrophages partially reversed these beneficial effects (44). Shen and colleagues investigated an M1-to-M2 phenotype switch in a model of UUO, and depletion of M2 macrophages attenuated EMT and subsequent fibrosis (14). In line with these (pre-)clinical findings, we discovered a transition in the intrarenal macrophage phenotype from HUS-induced AKI to CKD long-term state. Parallel to fibrosis development after 21 days, M2 macrophages revealed to be the predominant macrophage phenotype in the kidneys of mice with HUS.

In HUS pathology, leukocyte recruitment and activation contributes to disease progression and severity (21). Pro-inflammatory cytokine release by different cell types induced by Stx include IL-6, TNF-α, IL-1β, and GM-CSF, exacerbating renal injury and sensitivity to Stx (45, 46). In our 7-day model of HUS, we demonstrated the involvement of the classical and non-canonical NFκB pathway, including the renal abundance of the abovementioned cytokines and chemokines (47). Here, we prove that at least after 21 days, these cytokines were no longer released in the kidneys, supporting the hypothesis of an attenuated acute phase. Interestingly, the renal level of cytokines with monocyte/macrophage-chemotactic function was elevated 21 days after HUS induction, highlighting their involvement in CKD. The role of MCP-1 and RANTES in fibrogenesis was evaluated in a mouse model of UUO, in which both were increasingly expressed at sites of progressive fibrosis (48). In addition, Mansour and colleagues featured MCP-1 as a promising renal fibrosis biomarker for prediction and diagnosis (49). Besides its chemotactic capacity, the MCP-1/CCR2 axis has been associated with the transdifferentiation of macrophages to myofibroblasts in a model of Twist1-deficient mice with UUO in which the levels of MCP-1 and CCR2 and consequently renal fibrosis were reduced (50). MCP-5, possessing high similarity to MCP-1, revealed to have a pivotal role in pulmonary fibrogenesis, since cell-specific silencing of MCP-5 or neutralization attenuated pulmonary fibrosis (51, 52). CCL22 also seems to express a role in pulmonary fibrosis, with enhanced abundance in bronchoalveolar lavage fluid and serum of patients and in mice (53). Taken together, the chemokines that were highly abundant in the renal tissue of mice 21 days after HUS induction all possess the capacity to recruit especially macrophages contributing to fibrosis. As the corresponding receptors were proven to be of similar importance in the initiation of fibrotic signaling, their analysis would be of great interest.

## 5 Limitations of the study

Five individuals per group could be considered as low power; however, our results are explicit and comparing time and Stx concentration dependency was prioritized for the establishment of this Stx-induced CKD model. As mentioned earlier, the prevalence of CKD is influenced, among others, by sex, which has not been considered here, but will be taken into consideration in future studies. Additionally, CKD in humans is mainly detected by excretion changes in the urine. Since mice have major urinary proteins in the urine, pathologic proteinuria is hardly detectable due to permanently high non-pathological protein concentrations (54). Numerous advantages highlight the study of diseases in mice as the model organism. However, parts of the innate and adaptive immunity, such as toll-like receptor distribution and cytokine expression profiles, differ from mice to humans, based on evolutionary, epigenetic, and environmental influence [discussed in (55, 56)]. We report crucial similarities in our model compared to processes in humans; however, we still need to consider potential differences.

## 6 Conclusion

Clinical meta-studies describe the importance of long-term follow-up of patients with HUS due to symptoms developing 1–15 years after recovery from the initial illness, even in patients with mild disease without dialysis treatment (5, 8). We present, for the first time, a CKD model based on the bacterial toxemia of HUS. We proved that the acute phase attenuated after 21 days along with anemia transitioned from HUS-associated hemolytic to non-hemolytic microcytic anemia. In line with the histologically confirmed renal fibrosis, pro-fibrotic M2 macrophages and pro-fibrotic signaling were present in the kidneys of HUS mice.

Fibrosis without subsequent recurring damage, e.g., visual in plasma urea, represents the most critical period of time in the poorly understood transition from AKI to CKD. In comparison to other models, we here present the first representative CKD model in mice due to prior illness, without operational damage or artificial chemically induced nephrotoxicity but with a concentration-dependent severity. This model serves as a platform for the examination of the early underlying mechanisms of AKI-to-CKD transition and for therapeutic testing to prevent patients with HUS from developing CKD.

## Data Availability

The original contributions presented in the study are included in the article/**Supplementary Material**. Further inquiries can be directed to the corresponding author.
