# Supplementary material for: Transition from acute kidney injury to chronic kidney disease in a long-term murine model of Shiga toxin-induced hemolytic-uremic syndrome
Source: Front Immunol. 2024 Oct 10;15:1469353. doi: 10.3389/fimmu.2024.1469353 (PMC11499141; doi:10.3389/fimmu.2024.1469353)
Supplement: Supplementary file 1 [file DataSheet1.docx]

Supplementary Material

Transition from acute kidney injury to chronic kidney disease in a long-term murine model of Shiga toxin-induced hemolytic-uremic syndrome

Jamila Wegener^1,2^, Sophie Dennhardt^1,2^, Ivonne Loeffler^3^, Sina M. Coldewey^1,2,4*^

^1^Department of Anesthesiology and Intensive Care Medicine, Jena University Hospital, Jena, Germany

^2^Septomics Research Center, Jena University Hospital, Jena, Germany

^3^Department of Internal Medicine III, Jena University Hospital, Jena, Germany

^4^Center for Sepsis Control and Care (CSCC), Jena University Hospital, Jena, Germany

*** Correspondence:**Sina M. Coldewey
sina.coldewey@med.uni-jena.de

Keywords: Chronic kidney disease, acute kidney injury, animal model, fibrosis, anemia, hemolytic-uremic syndrome

**Supplementary Material List**

Supplementary Figure S1. Systemic abundance of proteins involved in resorption and storage of iron in mice with experimental hemolytic-uremic syndrome (HUS).

Supplementary Table S1. Commercial kits.

Supplementary Table S2. Primary antibodies for immunohistochemistry.

Supplementary Table S3. Secondary antibodies for immunohistochemistry.

**
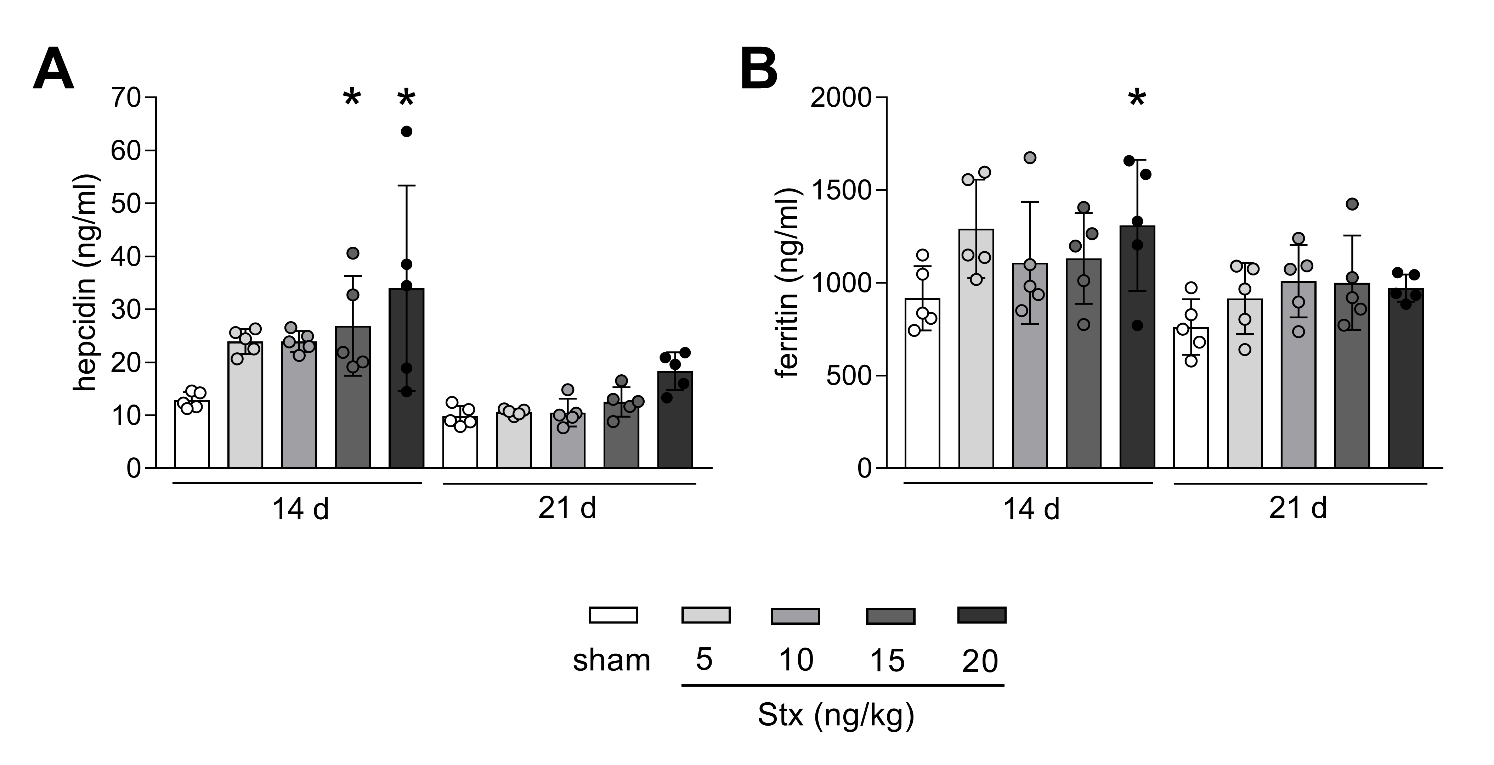
Supplementary Figure S1: Systemic abundance of proteins involved in resorption and storage of iron in mice with experimental hemolytic-uremic syndrome (HUS).** Plasma **(A)** hepcidin and **(B)** ferritin of sham mice or mice with experimental HUS 14 and 21 days after HUS induction (depicted Shiga toxin (Stx) concentrations). Data are shown as mean ± SD. n = 5. **P* < 0.05 compared to corresponding sham group (Two-way ANOVA and Dunnett’s multiple comparison test).

**Supplementary Table S1: Commercial kits**

| target | name | supplier | Cat. No. |
| --- | --- | --- | --- |
| **plasma NGAL** | LEGEND MAX™ Human NGAL (Lipocalin-2) ELISA Kit | BioLegend, USA | 443407 |
| **plasma urea** | Urea Assay kit | Abcam, UK | ab83362 |
| **plasma and renal EPO** | ELISA Kit for Erythropoietin (EPO) | Cloud-Clone Corp., USA | SEA028Mu |
| **LDH activity** | LDH Assay Kit / Lactate Dehydrogenase Assay Kit (Colorimetric) | Abcam, UK | ab102526 |
| **plasma ferritin** | Ferritin (Mouse) ELISA Kit | Abnova Corp., Taiwan | KA1941 |
| **plasma hepcidin** | ELISA Kit for Hepcidin (Hepc) | Cloud-Clone Corp., USA | SEB979Mu |

NGAL (neutrophil gelatinase-associated lipocalin), EPO (erythropoietin), LDH (lactate dehydrogenase), ELISA (Enzyme-linked immunosorbent assay)

Supplementary Table S2: Primary antibodies for immunohistochemistry

| target | blocking | Primary antibody | | | | |
| --- | --- | --- | --- | --- | --- | --- |
|  |  | name | supplier | Cat. No. | dilution factor | dilution buffer |
| **Ly6g** | 20% NRS in  1% BSA in  Tris buffer | monoclonal rat-anti-Ly6g | Abcam, UK | ab25377 | 1:100 | 1 % BSA in Tris buffer |
| **CD86** | 10 % NGS, 1% BSA, 0,4 % Tx100 in HBSS | B7-2/CD86 Antibody (BU63) | NovusBiologicals, USA | NBP2-25208 | 1:100 | 10 % NGS, 1% BSA, 0,4 % Tx100 in HBSS |
| **CD206** | 10 % NGS, 1% BSA, 0,4 % Tx100 in HBSS | Anti-Mannose Receptor antibody | Abcam, UK | ab64693 | 1:5000 | 10 % NGS, 1% BSA, 0,4 % Tx100 in HBSS |

Ly6G (lymphocyte antigen 6 family member G), CD206 (Cluster of Differentiation 206), CD86 (Cluster of Differentiation 86), NRS (normal rabbit serum), BSA (bovine serum albumin), HBSS (Hank’s buffered salt solution), Tx100 (Triton-X100), NGS (normal goat serum)

Supplementary Table S3: Secondary antibodies for immunohistochemistry

| name | supplier | Cat. No. | dilution factor | dilution buffer |
| --- | --- | --- | --- | --- |
| **biotinylated**  **anti-rat IgG (H+L)** | Vector Laboratories, USA | BA-4001 | 1:200 | 1 % BSA in Tris buffer |
| **biotinylated anti-rabbit IgG (H+L)** | Vector Laboratories, USA | BA-1000 | 1:200 | Tris buffer |
| **biotinylated anti-mouse IgG (H+L)** | Vector Laboratories, USA | BA-2001 | 1:200 | Tris buffer |
